# Asymptomatic Vaginal Candidiasis Complicated by Chorioamnionitis and Pelvic Abscess at Full-Term Delivery: A Case Report

**DOI:** 10.7759/cureus.79906

**Published:** 2025-03-01

**Authors:** Chihiro Nishino, Hiroshi Kawamura, Yuka Hattori, Makoto Orisaka, Yoshio Yoshida

**Affiliations:** 1 Department of Obstetrics and Gynecology, Sugita Genpaku Memorial Obama Municipal Hospital, Fukui, JPN; 2 Department of Obstetrics and Gynecology, University of Fukui, Fukui, JPN

**Keywords:** asymptomatic vaginal candidiasis, candida chorioamnionitis, case report, cesarean section (cs), pelvic abscess

## Abstract

Despite the high frequency of vaginal candidiasis during pregnancy, chorioamnionitis (CAM) is rarely caused by Candida* *species as the initiating organism, especially in full-term delivery. A 25-year-old Japanese woman was referred to our hospital at 34 weeks and two days of gestation and was diagnosed with vaginal candidiasis based on a vaginal culture examination. She delivered a newborn via cesarean section at 39 weeks and four days of gestation. The newborn had scattered erythematous small papules systemically. White patches were observed on the placental fetal surface and along the entire length of the umbilical cord. Amniotic fluid and cord blood cultures revealed the presence of *Candida albicans*. An antifungal agent was administered to the mother and newborn. Surgical drainage was required for the treatment of the intra-abdominal abscess of the mother. Placental pathology revealed histological CAM and funisitis, with a maternal inflammatory response of Stage 3/Grade 2 and a fetal inflammatory response of Stage 3/Grade 2. Periodic acid-Schiff stain demonstrated fungal yeasts in the amniotic membrane or near the surface of the umbilical cord.

Asymptomatic vaginal candidiasis can lead to maternal and neonatal development of disease due to CandidaCAM in the peripartum period even in full-term pregnancy. Especially in cesarean delivery, physicians should pay attention to postoperative pelvic abscess formation.

## Introduction

Chorioamnionitis (CAM) is an acute inflammation of the chorion, amnion, and placental membranes and is widely recognized as a major cause of spontaneous preterm birth. Typically, it results from an ascending polymicrobial bacterial infection following membrane rupture but can also occur with intact membranes [1]. Various bacteria, including Group B Streptococcus (GBS) and *Escherichia coli*, have been linked to CAM. Recently, Ureaplasma species and *Mycoplasma hominis*, commonly found in the lower genital tract, have been identified as major pathogens of CAM and its associated preterm birth [1].

Vaginal candidiasis is a common genital tract infection in pregnant women, with a reported prevalence of 13%-20% [2,3]. While genital pruritus and abnormal vaginal discharge are typical symptoms, the infection is often asymptomatic. Despite its high occurrence during pregnancy, Candida species rarely act as the initiating agent of CAM. However, a recent observational study suggests that perinatal outcomes of Candida CAM are often poor, primarily due to extreme preterm birth with a positive amniotic fluid culture [4]. Although genital tract infections can trigger spontaneous preterm birth through ascending infection and intrauterine inflammation [5], the relationship between asymptomatic vaginal candidiasis and Candida CAM remains unclear [3,6]. Additionally, there have been no reports of severe maternal adverse outcomes resulting from Candida CAM. However, Candida CAM can spread locally during delivery. In cesarean sections, surgical incisions, devitalized tissue, and hematoma create a favorable environment for fungal proliferation. Persistent infection or poor wound healing can subsequently lead to pelvic abscess formation.

This report presents a rare case of Candida CAM leading to maternal intra-abdominal Candida infection at 39 weeks of gestation in a patient with untreated asymptomatic vaginal candidiasis.

## Case presentation

A 25-year-old Japanese pregnant woman, G3P0A2, conceived spontaneously and had no history of gynecological, medical, or surgical conditions. She was referred to our hospital at 34 weeks and two days of gestation for perinatal care management. A human immunodeficiency virus antibody test performed at nine weeks of gestation was negative. Screening for glucose intolerance at nine and 24 weeks of gestation using blood glucose tests was negative. At the initial examination, no rash or redness was observed on the vulva, and a speculum examination showed scant white discharge. A culture of vaginal discharge and a rectal specimen for GBS screening on the same day were negative for GBS but positive for *Candida albicans*. No treatment was administered as she was asymptomatic.

**Figure 1:**
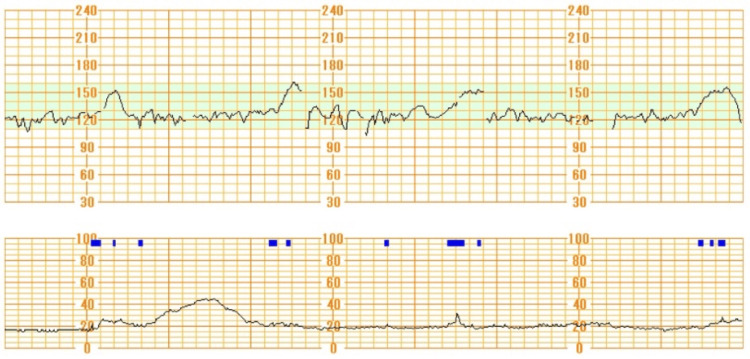
Cardiotocogram when deciding to perform cesarean section

At 39 weeks and two days, the patient was admitted for premature rupture of membranes (PROM). Her vitals were as follows: height 166 cm, body weight 78.2 kg, body temperature (BT) 37.0℃, blood pressure (BP) 120/78 mmHg, and heart rate (HR) 94 bpm. Amoxicillin was administered to prevent neonatal infection. The following day, labor was induced using prostaglandin E2 orally, 17 hours after PROM. After labor onset, antibiotics were changed to ampicillin sodium infusion. Six hours into labor, the cervix was 4 cm dilated, but the labor was almost arrested due to uterine inertia. Labor augmentation was considered, but the patient declined uterotonic drugs, requesting a cesarean section instead. Her preoperative vitals were BT 37.6℃, BP 115/74 mmHg, and HR 99 bpm. Blood tests showed a white blood cell (WBC) count of 16,420/μL (normal range: 3,100-8,400/μL) and C-reactive protein (CRP) of 4.3 mg/dL (normal range: <0.3 mg/dL). No abnormal fetal heart pattern was detected in the cardiotocogram (Figure 1).

**Figure 2:**
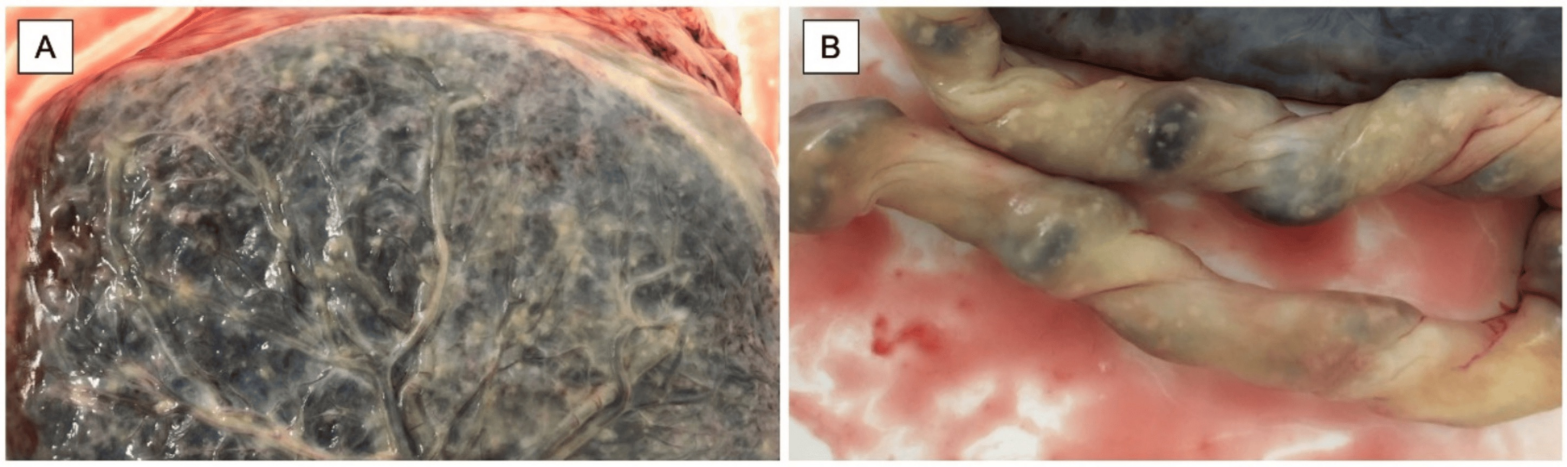
Macroscopic findings of (A) placenta and (B) umbilical cord, showing white patches spreading over the entire surface of the placenta and umbilical cord

During laparotomy, cysto-uterine fossa peritoneum, and lower transverse uterine incisions, no opacity was seen in the amniotic fluid. A female infant weighing 3,128 g was delivered with an Apgar score 8/10. White patches were observed on the placental fetal surface and along the entire length of the umbilical cord (Figures 2A, 2B). Suspecting an intrauterine infection, an intra-abdominal drainage tube was placed, and the cysto-uterine fossa peritoneum was not sutured. Intraoperative blood loss, including amniotic fluid, was 955 mL. The duration between PROM and delivery was approximately 43 hours.

On postoperative day (POD 2), maternal fever was elevated to 39℃. Blood tests revealed worsening inflammation (WBC 17,676/μL, CRP 18.0 mg/dL), suggesting intrauterine bacterial infection, prompting the addition of intravenous clindamycin (1,200 mg/day) to ampicillin sodium. Amniotic fluid and cord blood cultures collected during surgery indicated *C. albicans* infection without any other bacteria by Gram staining, leading to the addition of fluconazole infusion (800 mg/day). The postoperative maternal blood culture was negative, and β-D glucan was within the normal range (13.4 pg/mL; normal: <20 pg/mL). On POD 3, the intra-abdominal drainage tube was removed as the maternal fever had resolved. On POD 4, BT reduced to 36°C, leading to the discontinuation of ampicillin and clindamycin while continuing antifungal therapy.

**Figure 3:**
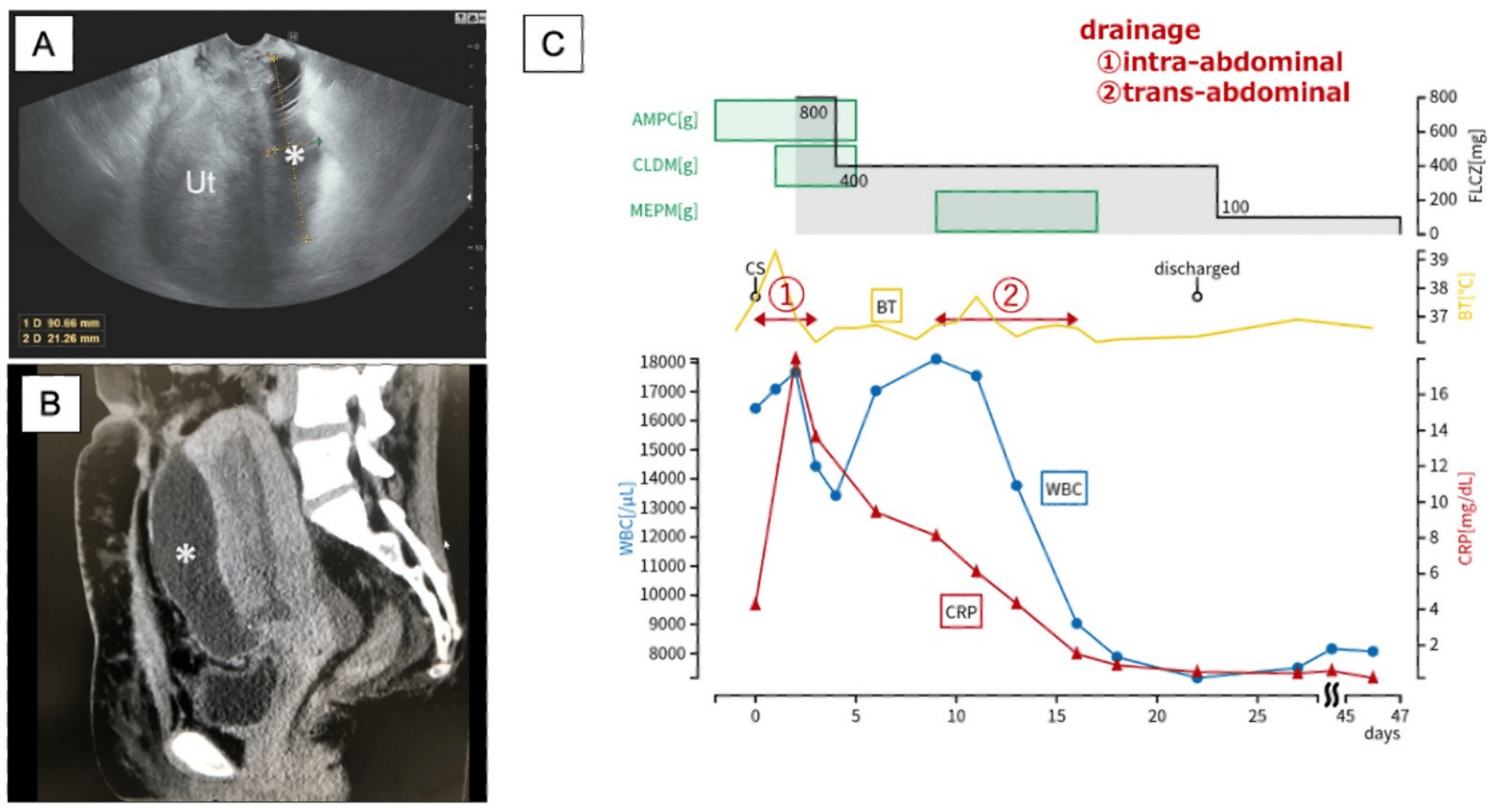
(A) Sonographic findings of pelvic abscess on the anterior surface of the uterus on POD 6. (B) Computed tomography of the pelvis on POD 9. (C) Therapeutic timeline Asterisks show the abscess Ut: uterus; AMPC: ampicillin; CLDM: clindamycin; MEPM: meropenem; FLCZ: fluconazole; CS: cesarean section; BT: body temperature; WBC: white blood counts; CRP: C-reactive protein; POD: postoperative day

On POD 6, a transvaginal ultrasound demonstrated a fluid accumulation approximately 9 cm anterior to the uterus (Figure 3A). The patient remained stable, so antifungal therapy was continued. On POD 9, blood tests revealed WBC 18,120/μL, CRP 8.1 mg/dL, and β-D glucan 15.7 pg/mL. Contrast-enhanced computed tomography revealed a large pelvic abscess (12.4 x 4 x 8.3 cm) extending from the uterine incision site to the anterior surface of the uterus (Figure 3B). Thus, transabdominal ultrasound-guided drainage was performed. The culture of the drained fluid revealed a high load of C. albicans without any other bacteria by Gram staining. Intra-abdominal drainage was completed on POD 15.

On POD 22, blood tests showed improvement (WBC 7,160/µL and CRP 0.5 mg/dL). Transvaginal ultrasonography showed that the abscess had reduced to 5 cm, fluconazole was switched from intravenous to oral administration (100 mg/day), and the patient was discharged. On POD 46, the inflammatory response normalized, the abscess resolved, and oral fluconazole was discontinued. Figure 3C demonstrates the therapeutic timeline, and Table 1 shows maternal hematological data, including WBC, CRP, and β-D glucan before and after cesarean section.

**Table 1:** Maternal hematology data of WBC, CRP, and β-D glucan WBC: white blood count; CRP: C-reactive protein; CS: cesarean section; POD: postoperative day

Parameter	Normal range	Day 0 (before CS)	POD 2	POD 3	POD 6	POD 9	POD 16	POD 22	POD 46
WBC	3,100-8,400/μL	16,420	17,676	14,430	17,030	18,120	9,030	7,160	8,070
CRP	<0.3 mg/dL	4.3	18	13.6	9.4	8.1	1.5	0.5	0.18
β-D glucan	<20 pg/mL	-	13.4	-	-	15.7	-	-	-

**Figure 4:**
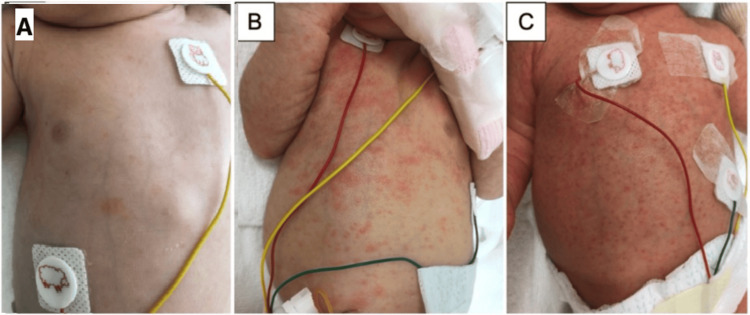
Skin rashes of the neonatal trunk area. (A) Several small pale erythemas on the anterior chest (day 1). (B) Papules spread over the entire trunk (day 2). (C) Several generalized small reddish papules over erythematous area (day 4)

The newborn was presented with scattered systemic erythematous small papules immediately after birth, and bacterial infection was suspected from yellow staining and leukoplakia of the umbilical cord. Consequently, ampicillin sodium (0.3 g/day) and Cefotaxime sodium (300 mg/day) were initiated. The newborn was in stable condition. Blood tests at the start of antibiotics showed WBC 16,050/μL (normal range: 19,600 ± 5,600/μL), neutrophil count 8,290/μL (immature-to-total-neutrophil ratio 0.4), CRP 0.4 mg/dL (normal range: 0.03 ± 0.09 mg/dL), and IgM 6 mg/dL (normal range: 0.03 ± 0.09 mg/dL). Umbilical culture was positive for *C. albicans*, while blood and nasal cultures were negative. On day 1, the CRP increased to 1.9 mg/dL, and skin rash was exacerbated, showing exudation (Figure 4A). On day 2, *C. albicans* was detected in bacterial cultures of amniotic fluid, cord blood, and neonatal umbilical surface obtained during cesarean section. Fluconazole (12 mg/kg/day at first and 6 mg/kg/day thereafter) was started, and imidazole antifungal was applied to the skin rash (Figure 4B). The skin rash became blisters (Figure 4C), but CRP decreased to 0.38 mg/dL by day 4. No intraocular inflammation was observed. The patient was switched to oral fluconazole (3 mg/kg/day) on day 10, and medication was completed by day 32. No neurological abnormalities were observed by 12 months of age.

**Figure 5:**
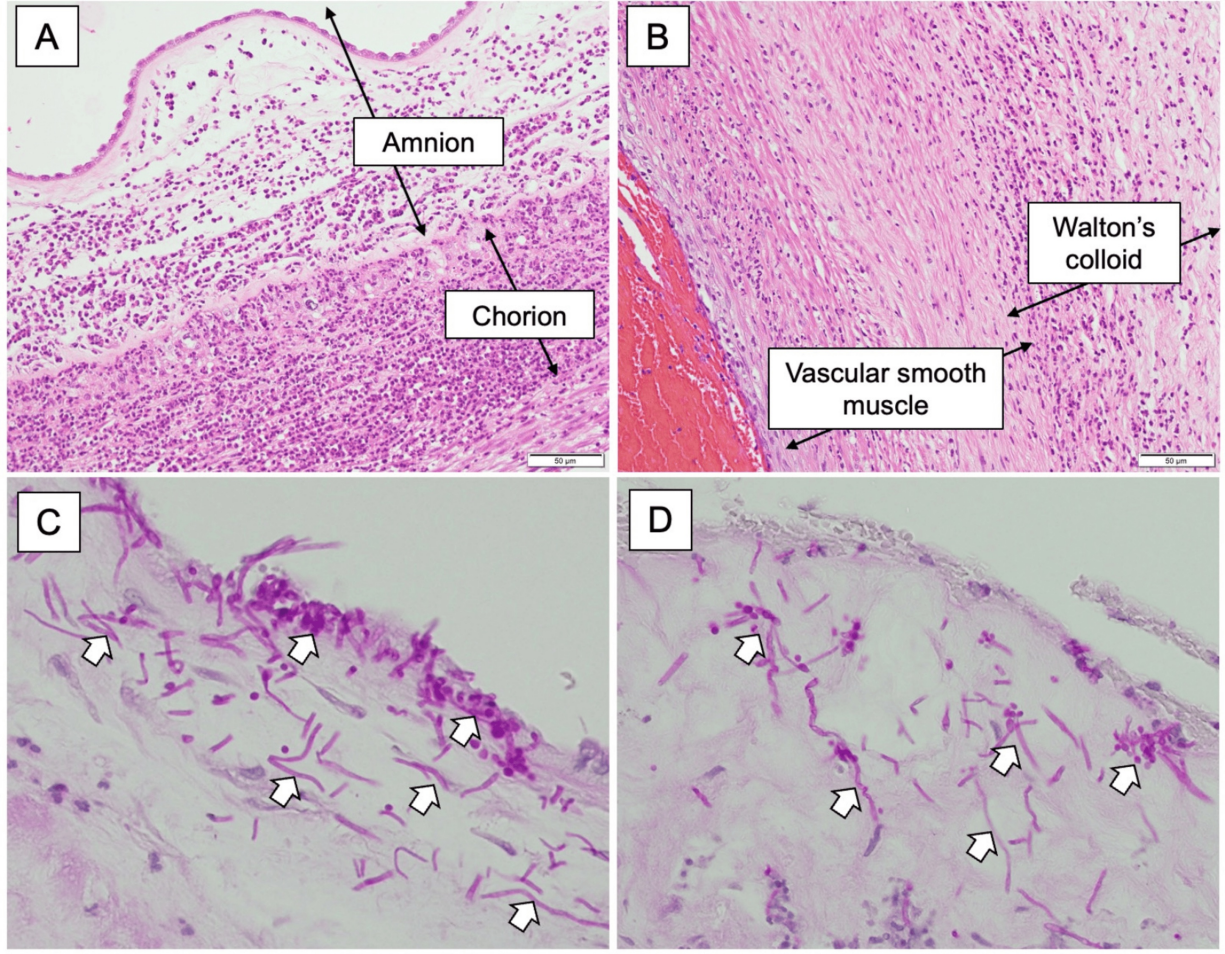
Microscopic pathological findings of placenta and umbilical cord. (A) Neutrophilic infiltration extends to the amnion, indicating histological CAM. (B) Neutrophil infiltration extends beyond the vascular smooth muscle to Walton's colloid. PAS stain demonstrated fungal yeasts (reddish purple mycelia) in the amniotic membrane (C) or near the surface of the umbilical cord (D) White arrows indicate fungal yeasts CAM: chorioamnionitis; PAS: periodic acid-Schiff

According to the Amsterdam criteria, histopathological examination of the placenta and umbilical cord revealed a maternal inflammatory response of Stage 3/Grade 2 and a fetal inflammatory response of Stage 3/Grade 2 (Figures 5A, 5B). Periodic acid-Schiff staining demonstrated the presence of fungal yeasts in the amniotic membrane and near the umbilical cord surface (Figures 5C, 5D).

## Discussion

This case shows the potential for untreated asymptomatic vaginal candidiasis to develop to Candida CAM, leading to postpartum maternal pelvic abscess, even in a full-term pregnancy. To our knowledge, this is the first reported case of maternal intra-abdominal abscess formation caused by Candida CAM, requiring transabdominal drainage.

Candida CAM is very rare, with a reported incidence of 0.3%-0.5% of all deliveries [4,7]. Reported risk factors for Candida CAM include intrauterine contraceptive devices (IUD), in vitro fertilization (IVF), maternal diabetes mellitus, and amniocentesis or cervical cerclage during pregnancy [4]. However, the patient in this case had no known risk factors. Clinical or histological CAM is detected in 40%-70% of preterm births with PROM or spontaneous labor [1]. Maki et al. reported nine cases of Candida CAM among approximately 2,700 deliveries in a 10-year single-center study, all of which were preterm, and seven of them resulted in extremely preterm infants [4]. In contrast, Obermair et al. reported an even rarer case of Candida CAM causing fetal death and stillbirth at 37 weeks of gestation, with the presence of histological multiple umbilical cord abscesses [8].

Placental pathology in this case showed that Candida organisms were concentrated on the surface of the umbilical cord and amnion (Figures 4C, 4D), suggesting an ascending intrauterine infection from the vagina following membrane rupture. However, reports from in vitro studies showed that *C. albicans* can also pass through intact amniotic membranes [9,10], indicating the possibility that intrauterine infection may have occurred before the rupture of the fetal membrane.

In this case, *C. albicans* was detected in the GBS screening culture at 34 weeks of gestation, but no treatment was administered because the patient was asymptomatic. A recent systematic review and meta-analysis found no association between asymptomatic vaginal Candida colonization and preterm delivery or other perinatal or maternal complications and no evidence of a benefit from antimicrobial therapy [6]. However, this research did not examine the relationship between asymptomatic vaginal Candida colonization and the risk of Candida CAM. Thus, further study is needed to verify the effectiveness of vaginal candidiasis treatment against ascending Candida infection and the subsequent development of Candida CAM.

In the current era, pelvic abscess formation following cesarean section is rare due to the widespread use of perioperative antimicrobial agents. While prophylactic administration of antibiotics has been reported to prevent bacterial infections associated with cesarean section [11], antifungal agents are not commonly used perioperatively, even in the presence of vaginal candidiasis. In the present case, the abscess formed extending from the uterine incision site to the anterior surface of the uterus was successfully drained under transabdominal ultrasound guidance. Percutaneous abscess drainage is a less invasive and more effective treatment for pelvic abscesses than open drainage. As performed in this case, an anterior percutaneous approach guided by ultrasound is a relatively simple and sufficient method for draining abscesses existing from the cysto-uterine fossa to the anterior surface of the uterus. Nonetheless, reports have shown that when the abscess is located posterior to the uterus, an anterior approach may be difficult, and other methods, such as ultrasound-guided transvaginal or radiologically guided paracoccygeal-infragluteal approach, may be required [12]. Thus, the appropriate drainage approach for pelvic abscesses should be guided by imaging and adapted to the size and location of the abscess.

Currently, there are limited reports on the peripartum risks of maternal deterioration due to Candida infection. A literature review was conducted by searching PubMed for English case reports or case series documented through March 3, 2024, using the search terms ''candida'' AND ''chorioamnionitis''. A total of 51 articles containing 63 maternal cases were obtained. Among them, maternal peripartum candidemia was developed in eight cases, mainly caused by *Candida glabrata* (four of eight), and only one case was attributed to *C. albicans*. Details of the eight cases, including the current one, are shown in Table 2 [13-20]. Barth et al. reported septic shock in a mother who conceived while using an IUD, with intrauterine fetal death due to *Candida tropicalis* infection at 19 weeks of gestation [18]. From the literature, no reports of a case requiring drainage therapy for peripartum pelvic abscess due to Candida CAM have been documented. In this case, the patient had a history of recurrent artificial abortions with intrauterine surgeries. While IVF has been considered a risk factor for Candida CAM, previous repeated intrauterine surgeries may play a role in its development. However, it remains unclear whether intrauterine surgeries involving physical damage to the endometrium or cervical procedures predispose patients to fungal infection.

**Table 2:** A summary of nine cases (eight case reports and ours) complicated by peripartum candidemia AC: amniocentesis; DM: diabetes mellitus; GDM: gestational diabetes mellitus; PROM: premature rupture of membrane; GA: gestational age; ND: no data; IVF: in vitro fertilization; IUD: intrauterine device; VD: vaginal delivery; CS: cesarean section

No	Study	Age	Conception	Gestation	AC	Cerclage	DM/GDM	Vaginal candidiasis	PROM	GA at delivery	Delivery mode	Bacteria	Perinatal death	Maternal severe infection	Maternal death
1	Shiro et al. [13]	28	IVF	Singleton	-	-	-	Detected at PROM	18 weeks	18 weeks	VD	C. glabrata	Yes	Candidemia	-
2	Yusoff et al. [14]	ND	IVF	Twin	-	-	ND	-	17 weeks	19 weeks	VD	C. glabrata	Yes	Candidemia	-
3	Akhanoba et al. [15]	41	IVF	Twin	-	-	-	18 weeks, untreated	20 weeks	23 weeks	VD	C. glabrata	Yes	Candidemia	-
4	Jackel and Lai [16]	39	IVF	Twin	Yes	-	-	21 weeks, untreated	22 weeks	24 weeks	-	C. glabrata	Yes	Candidemia	-
5	Pineda et al. [17]	34	IVF	Twin	-	-	-	ND	29 weeks	29 weeks	CS	C. kefyr	-	Candidemia	-
6	Barth et al. [18]	35	Spontaneous with IUD	Singleton	-	-	-	-	ND	19 weeks	VD	C. tropicalis	Yes	Candidemia, septic shock	-
7	Huang et al. [19]	35	Spontaneous	Singleton	-	-	-	-	-	27 weeks	-	C. albicans	-	Candidemia	-
8	DiLorenzo et al. [20]	32	IVF	Twin	Yes	-	-	-	-	17 weeks	VD	C. lusitaniae	Yes	Candidemia	-
9	Our study	25	Spontaneous	Singleton	-	-	-	34 weeks, untreated	39 weeks	39 weeks	CS	C. albicans	-	Pelvic abscess and drainage	-

In this case, we administered antimicrobials to prevent intrauterine infection associated with PROM; however, the possibility that this prophylactic use of antimicrobials may have exacerbated the fungal infection cannot be completely ruled out. Therefore, physicians should refrain from unnecessary use of antimicrobials, carefully weighing the risks and benefits of antimicrobial administration in peripartum pregnant women. Labor induction using prostaglandin E2 was initiated 17 hours after PROM rather than immediately due to a limited number of available providers to ensure patient safety. This management approach remained consistent with the standard of care in Japan. The decision to use prostaglandin E2 was based on poor cervical maturation and the expectation that it would promote cervical ripening. Appropriate clinical management for PROM is crucial in preventing severe infections. Therefore, flexibility in determining the timing of induction of labor and selecting a uterotonic agent based on the patient's condition is essential. In this case, considering the findings of placenta and umbilical cord at delivery, along with a history of vaginal candidiasis, consultation with an infectious disease specialist regarding the administration of antifungal agents could have been warranted.

## Conclusions

Asymptomatic vaginal candidiasis can lead to maternal and neonatal Candida infection due to CAM in the peripartum period, even in full-term pregnancies. In cesarean delivery, postoperative pelvic abscess formation should be considered as a potential complication following Candida CAM. Further studies are needed to explore the correlation between asymptomatic vaginal candidiasis and Candida CAM, and to provide effective therapeutic methods to address the risks of ascending infections during pregnancy. Appropriate use of antimicrobials is important in preventing fungal infections, and their use in peripartum pregnant women should be carefully determined, considering the risks and benefits of administration.
